# A Robust AQbD-Based RP-HPLC Method for Simultaneous Quantification of Acyclovir and Doxepin Hydrochloride in Bulk and Topical Cream Formulation

**DOI:** 10.3390/molecules31193565

**Published:** 2026-10-07

**Authors:** Akshay Kumar, Mohit Kumar, Abhishek Saini, Mohit Agrawal, Urvashi Khatri, Neeraj Choudhary, Dinesh Kumar, Ammar Cherif

**Affiliations:** 1 Chitkara College of Pharmacy, Chitkara University, Rajpura 140401, India; akranaut2000@gmail.com (A.K.); mohit.5167@chitkara.edu.in (M.K.); 2 Ayush Herbs Pvt. Ltd., Kangra 176047, India; urvashimaria@gmail.com; 3 School of Medical & Allied Sciences, K.R. Mangalam University, Gurugram 122103, India; mohitagrawalmohu@gmail.com; 4 GNA School of Pharmacy, GNA University, Phagwara 144401, India; dr.neerajchoudhary@gmail.com (N.C.); dineshpotlia123@gmail.com (D.K.); 5 Department of Biology, College of Science, Qassim University, Buraydah 51452, Saudi Arabia

**Keywords:** AQbD, high-performance liquid chromatography, topical drug delivery

## Abstract

The present study describes the development and validation of a simple, rapid, and reliable RP-HPLC method for the simultaneous estimation of acyclovir and doxepin hydrochloride in bulk and topical cream formulation using an Analytical Quality by Design (AQbD) approach. The combination of acyclovir, an antiviral agent, and doxepin, an antipruritic and anti-inflammatory drug, offers a combined topical approach for the management of herpes-associated skin conditions. The method development was guided by a predefined Analytical Target Profile (ATP), followed by systematic risk assessment to identify critical quality attributes (CQAs) and critical method parameters (CMPs). The central composite design (CCD) was employed to optimize key chromatographic variables, including mobile phase composition and flow rate. The chromatographic separation was achieved on a C18 column using an isocratic mobile phase of acetonitrile and water (40:60 *v*/*v*) at a flow rate of 1.0 mL/min, with UV detection at 240 nm. The retention times were found to be 2.58 min for acyclovir and 4.64 min for doxepin hydrochloride, indicating efficient separation with good peak symmetry. The method was validated according to ICH Q2(R2) guidelines and demonstrated excellent linearity over the concentration ranges of 2–10 µg/mL (acyclovir) and 12–28 µg/mL (doxepin hydrochloride), with correlation coefficients greater than 0.997. The method exhibited high precision (%RSD < 2%), accuracy (recovery within 99–101%), and good sensitivity with low LOD and LOQ values. The robustness studies confirmed the reliability of the method under small, deliberate variations in chromatographic conditions. The developed cream formulation showed uniform drug content and satisfactory in vitro drug release, with a sustained release profile reaching approximately 96% over 24 h. In conclusion, the proposed AQbD-based RP-HPLC method is suitable for routine quantitative analysis and quality control of acyclovir and doxepin hydrochloride in combined topical formulations.

## 1. Introduction

**Figure 1:**
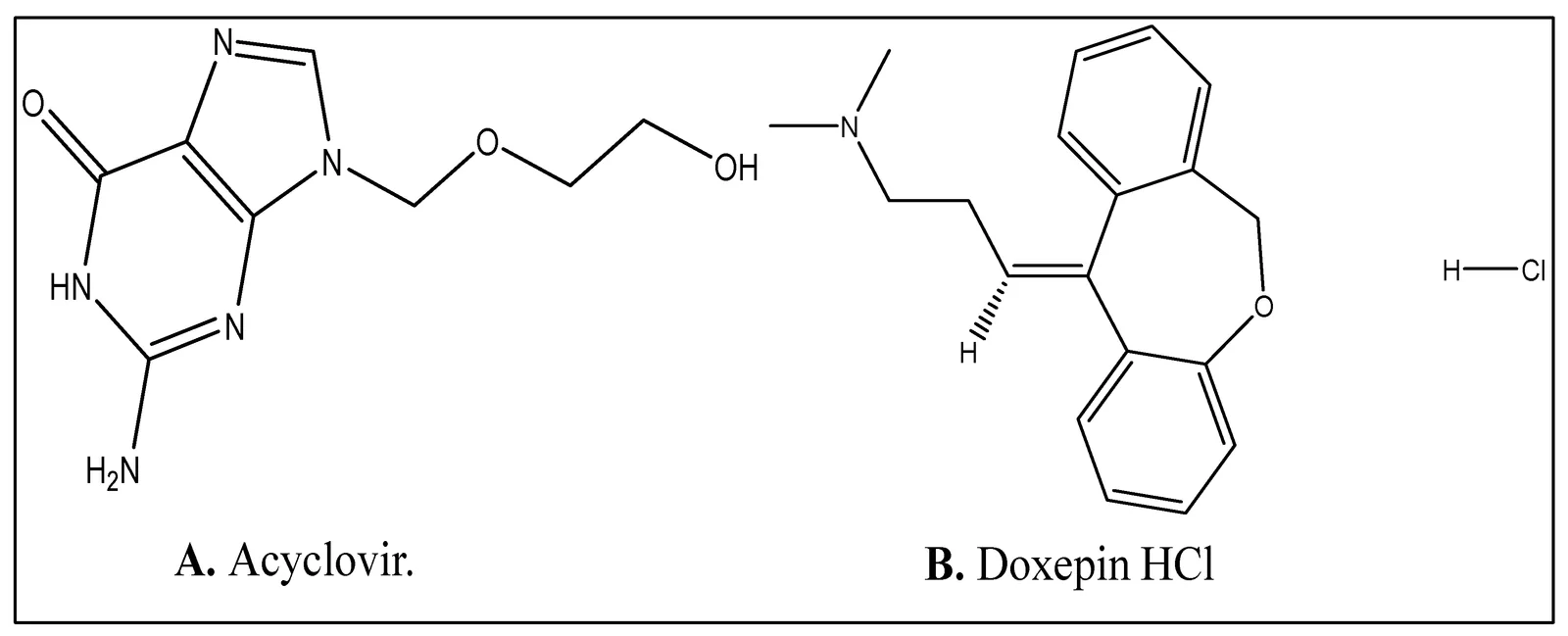
The structure of (**A**) Acyclovir (**B**) Doxepin HCl.

The effective management of herpes-related dermatological conditions is a critical therapeutic issue, as viral replication and inflammatory responses are dual agents. Acyclovir is a synthetic antiviral drug of the purine (guanine) nucleoside analogue class, chemically known as 2-amino-1,9-dihydro-9-[(2-hydroxyethoxy) methyl]-6H-purin-6-one with the molecular formula C_8_H_11_N_5_O_3_ (Figure 1A) [1]. It has a strong antiviral effect, selectively inhibiting viral DNA polymerase after intracellular activation by viral thymidine kinase. It results in the untimely arrest of the process of elongating the chain of viral DNA, thus, in effect, inhibiting the process of viral replication at minimal toxicity to the host cells [2]. Doxepin is a synthetic drug, a tricyclic antidepressant (TCA) [3]. It is chemically described as (E/Z)-3-(dibenzo[b,e]oxepin-11(6H)-ylidene)-N,N-dimethylpropan-1-amine, with the molecular formula C_19_H_21_NO (Figure 1B) [4]. It is well known to have a strong antipruritic and anti-inflammatory effect, which is mainly mediated by strong antagonism of histamine H1 receptors, but also by modulation of serotonin and norepinephrine reuptake [5,6]. Doxepin administered topically will symptomatically relieve itching, erythema, and discomfort commonly experienced by patients with viral skin infections [7].

The combination of acyclovir and doxepin in a single topical formulation would provide a rational, combined topical approach by simultaneously targeting the underlying viral etiology and the associated inflammatory manifestations. This type of combination therapy not only increases the overall effectiveness of treatment but also improves patient compliance due to the necessity of using multiple medications. The need to develop combination formulations, however, requires a reliable, sensitive, and robust analytical method to simultaneously estimate the two active pharmaceutical ingredients in both bulk and cream formulations. High-Performance Liquid Chromatography (HPLC) has become the method of choice in the analysis of pharmaceuticals because of its high level of resolution, sensitivity, and reproducibility [8]. Even though several HPLC methods have been reported for the individual estimation of acyclovir and doxepin, these methods often require complex gradient systems, longer run times, or are not specifically designed for simultaneous estimation in combined topical formulations. In addition, few studies have been conducted to optimize analytical conditions in a systematic, science-based manner, which is essential for method robustness and compliance with regulations.

Sankar et al., developed an RP-HPLC method for Acyclovir using a C18 column with water: methanol (50:50 *v*/*v*), showing a retention time of 3.077 min at 250 nm. The method exhibited excellent linearity (2–10 µg/mL, r^2^ = 0.999), high precision (%RSD < 2), and good sensitivity (LOD 0.247 µg/mL, LOQ 0.74 µg/mL), and was validated as per ICH guidelines for bulk and tablet analysis [9]. Chundur et al., developed and validated UV spectrophotometric and RP-HPLC methods for the simultaneous estimation of Acyclovir and Zidovudine using a mobile phase of water: methanol: acetonitrile (40:25:35 *v*/*v*) with 2% TEA, achieving retention times of 3.36 and 4.43 min, respectively. The method showed excellent linearity (2–10 µg/mL, R^2^ > 0.996), good sensitivity (LOD 0.42–0.51 µg/mL; LOQ 1.29–1.55 µg/mL), and was validated as precise, accurate, and suitable for routine quality control analysis [10]. Aboelezz et al., developed a simple and eco-friendly RP-HPLC method for the estimation of Acyclovir in human plasma using protein precipitation with acetonitrile and a mobile phase of water: methanol (97:3 *v*/*v*), achieving a retention time of ~15 min at 254 nm. The method showed good linearity (0.70–60 mg/L, r^2^ > 0.999) with high accuracy and precision (<7%), making it suitable for clinical monitoring of acyclovir levels [11]. Genç et al., developed a sensitive RP-HPLC method for the simultaneous determination of Acyclovir along with preservatives (methylparaben and propylparaben) using a C18 column with methanol and ortho-phosphoric acid as mobile phase. The method showed excellent linearity (R^2^ = 0.9979–0.9997), high accuracy (99.4–101.3%), precision (RSD ≤ 2%), and was successfully applied for analysis in tablet, cream, and suspension formulations [12]. Rajesh et al., developed a stability-indicating RP-HPLC method for Doxepin using a C18 column with methanol: acetonitrile: buffer (40:30:30 *v*/*v*/*v*), achieving a retention time of 2.92 min at 254 nm. The method showed good linearity (10–50 µg/mL, R^2^ = 0.9974), high accuracy (97.67–101%), and sensitivity (LOD 0.40–0.50 µg/mL), and was validated as precise and reliable for bulk and formulation analysis [13]. Nagalaxmi et al., developed an RP-HPLC method for the simultaneous estimation of Doxepin isomers using a phenyl column with phosphate buffer: methanol (60:40 *v*/*v*), achieving retention times of 6.989 and 7.936 min at 254 nm. The method showed good linearity (49.87–149.62 µg/mL), high accuracy (98–102%), and acceptable system suitability parameters, validating its reliability for bulk and dosage form analysis as per ICH guidelines [14].

The concept of Analytical Quality by Design (AQbD) has gained significant attention as a modern, systematic framework for analytical method development [15]. The focus of AQbD is on predefined objectives, risk assessment, critical method parameters (CMPs) and critical quality attributes (CQAs). Design of Experiments (DoE) and especially Central Composite Design (CCD) are used to efficiently explore the design space and to optimise chromatographic conditions in a statistically significant manner using a minimum number of experimental trials [16]. This will not only improve the robustness and reliability of the methods but also ensure that their performance remains consistent across different conditions, aligning with current regulatory expectations. Hence, the current investigation aims to design and validate a robust RP-HPLC method to simultaneously estimate acyclovir and doxepin in bulk and cream formulations within an AQbD-based strategy. The method was optimised using CCD to systematically evaluate key variables and determine the optimal chromatographic conditions. The suggested approach is likely to provide quick, accurate, and reproducible analytical method for regular quality control and pharmaceutical testing of the developed combination formulation.

## 2. Experimental Studies

### 2.1. Material Used

Acyclovir was acquired in Parkleton Pvt. Ltd. (Maharashtra, India), and Doxepin hydrochloride was purchased in Yarrow Chem Products (Mumbai, Maharashtra, India). Acetonitrile (ACN), water (HPLC grade), white petroleum jelly, white beeswax, stearic acid, Tween 80, Span 80, methyl paraben, propylene glycol, and titanium dioxide were purchased from Loba Chemie Pvt Ltd. (Mumbai, Maharashtra, India). All other chemicals and solvents employed in the study were of analytical or HPLC grade.

### 2.2. Table of Instruments and Apparatus Used

The chromatographic analysis was performed using a High-Performance Liquid Chromatography (HPLC) system (Shimadzu Corporation, Japan) equipped with a pump (LC-20AD), a UV-Visible detector (SPD-20A), a manual sampler, and the Winchrom program. Separation was achieved on a reversed-phase C18 column (Orbit 100 C18, 150× 4.6 mm; 3.5 µm). The preparation and analysis of the samples were done through a water-bath sonicator.

### 2.3. Choosing Analytical Wavelength

The analytical wavelength for the RP-HPLC method was selected based on the UV absorption spectra of Acyclovir and Doxepin hydrochloride, obtained using a UV-visible spectrophotometer. A working concentration of 10 µg/mL Acyclovir was prepared by diluting a 1000 µg/mL stock solution of Acyclovir in methanol with water. Likewise, a stock solution of Doxepin HCl (1000 µg/mL) was prepared in methanol and diluted to 10 µg/mL with water. The solutions were scanned in the UV region, between 200 and 400 nm, against an appropriate blank. The overlapping spectra of the two drugs were analysed, and the wavelength at which both drugs exhibited sufficient absorbance and good sensitivity with minimal interference was selected as the analytical wavelength for subsequent HPLC analysis.

### 2.4. Analytical Quality by Design-Based Method Development

The systematic AQbD approach was used to develop an analytical method for the simultaneous estimation of Acyclovir and Doxepin HCl in accordance with ICH Q14 guidelines. AQbD focuses on a risk- and science-based process of developing sound analytical procedures and having preset performance standards.

#### 2.4.1. Analytical Target Profile

The ATP was created to specify the intended use and the performance characteristics required for the analytical method. The ATP targets accurate and precise quantification of each of the two analytes in the cream formulation with sufficient specificity and sensitivity. The technique was tailored to deliver credible results with satisfactory accuracy, precision, and resolution within a limited analysis time. In compliance with ICH Q14, ATP was used as a starting point in method development, analytical condition selection, and the development of a validation strategy [17]. To ensure the developed method can be utilised for its intended purpose throughout its lifecycle, it is important to keep it fit for that purpose. Table 1 lists the ATP of the HPLC method.

#### 2.4.2. Identification of Critical Analytical Attributes and Method Variables (CQA, CMA, and CPP)

A systematic identification of critical analytical properties and method variables was conducted to develop a robust, reliable RP-HPLC method for simultaneous estimation of Acyclovir and Doxepin hydrochloride. According to the defined ATP, CQAs were identified as retention time, peak area response, resolution between analytes, tailing factor, and theoretical plate count since these directly influence the accuracy, precision and specificity of the method. Also, the Critical Material Attributes (CMAs), such as the composition and quality of the mobile phase, solvent purity, and column characteristics, including the stationary phase and particle size, were identified as important factors influencing chromatographic behaviour. Moreover, the Critical Process Parameters (CPPs), including flow rate, detection wavelength, injection volume, and column temperature, were identified depending on prior knowledge, scientific knowledge, and preliminary testing, as they would have a severe effect on the CQAs [18]. The overall identification and knowledge of these parameters facilitated the development of a method optimisation and control strategy based on science and risk, ensuring consistent method performance and control strategy throughout the analytical lifecycle. Table 2. showed risk-assessed key method variables that influence ATP.

#### 2.4.3. Quality Risk Assessment

**Figure 2:**
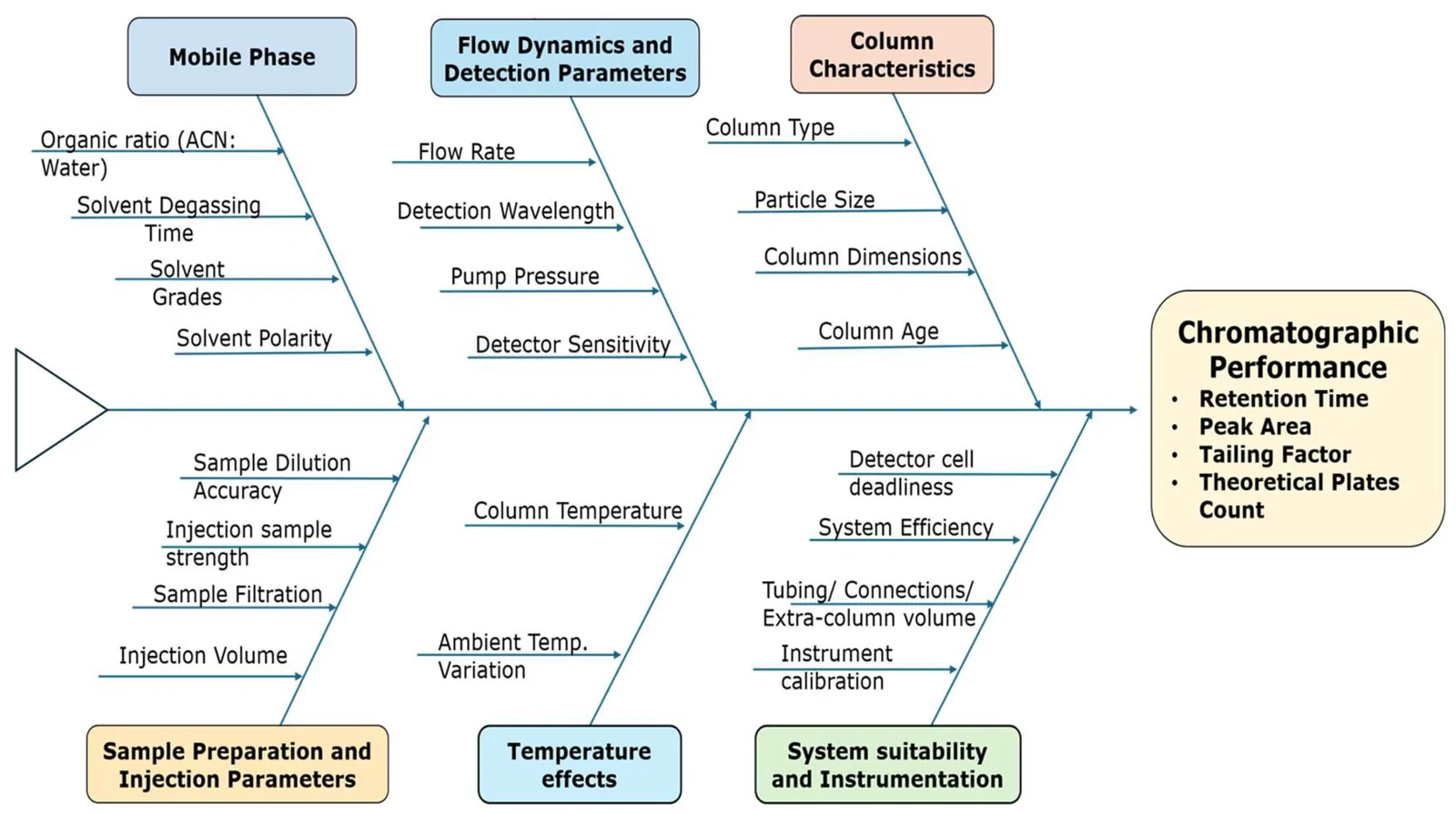
Ishikawa Fishbone diagram for the developed HPLC method in accordance with ICH Q2 (R2).

A preliminary risk assessment was carried out in accordance with the principles of to identify potential method variables that may influence the performance of the RP-HPLC method for simultaneous estimation of Acyclovir and Doxepin hydrochloride. All potentially significant analytical variables, such as mobile phase composition, flow rate, detection wavelength, column characteristics, and injection volume, were considered at this stage without prior classification of their criticality. The evaluation was done on scientific knowledge, the literature evidence, and a provisional understanding of chromatographic behaviour. The possible influence of these variables on CQAs, including retention time, resolution, peak area, and tailing factor, was systematically determined. The possible causes of variability were illustrated using an Ishikawa (fishbone) diagram (Figure 2), and a risk estimation matrix (Table 3) was used to classify the variables into low (green colour), medium (yellow colour), and high risk (Red colour) categories. In the risk matrix, red indicates a high-risk or critical impact, suggesting that small variations in the respective parameter may significantly affect chromatographic performance and therefore require stringent control and subsequent optimization. Yellow represents a moderate risk, indicating that the parameter may exert a measurable influence on the response and should be monitored within defined operational limits, whereas green denotes a low-risk impact, suggesting minimal influence on the corresponding CQA under normal analytical conditions. This preliminary risk assessment enabled the systematic prioritization of method variables for further experimental evaluation. Accordingly, the identified factors were subsequently subjected to experimental screening to establish their relative significance and criticality before final optimization of the chromatographic method [19,20].

According to the initial risk assessment, which included the Ishikawa fishbone diagram and the risk matrix developed in accordance with the ICH Q2(R2) guidelines, a factor screening was performed to identify the most influential method parameters [21]. The evaluation revealed that mobile phase composition and flow rate significantly affected CQAs, particularly retention time, resolution, and theoretical plates, and highlighted their robust influence on overall chromatographic performance. Based on this, these parameters were selected as CMPs and were subsequently considered for optimisation. Factors that had a medium or low impact were also considered less critical at this stage. This methodology ensured that experimental activities focused on the most important variables influencing method performance.

#### 2.4.4. Optimisation Study

Optimisation of chromatographic conditions was performed using Response Surface Methodology (RSM) with Design-Expert (Version 13). To examine the impact of two independent variables that included flow rate (0.8–1.2 mL/min) and mobile phase composition (ACN: Water, 30:70–50:50 *v*/*v*) on the chromatographic performance of the analytes was used. The CCD comprised 13 experimental runs, five of which were replicates at the centre point, to ensure method reproducibility and to estimate pure error, as shown in Table 4. Each experimental run was performed using the prepared mobile phase, prepared according to the given ratio, filtered through a membrane filter, and degassed before use. The chromatography system was equilibrated with the chosen conditions, and the standard solutions of the analytes were injected. All the runs were done under controlled conditions, with the levels of the chosen factors varied according to the design matrix. The chromatographic responses obtained after each run consisted of the retention time (RT) and tailing factor (TF) for Acyclovir and Doxepin HCl. These responses were considered key parameters for measuring the system’s performance and the efficiency of the approaches. All experiments were done in an unsystematic order to limit systematic bias. Analysis of the experimental data was performed using Design-Expert software via multiple regression. To explain the relationship between the independent variables and responses, a second-order poly model was fitted:

$$Y=\beta_{0}+\beta_{1}X_{1}+\beta_{2}X_{2}+\beta_{3}X_{1}^{2}+\beta_{4}X_{2}^{2}+\beta_{5}X_{1}X_{2}$$

The relationship between the independent variables and the selected chromatographic responses was described using a second-order polynomial equation. In the model, Y represents the predicted response or dependent variable, corresponding to the selected CQA, whereas $\beta_{0}$ denotes the intercept representing the predicted response at the centre point of the experimental domain. The coefficients $\beta_{1}$ and $\beta_{2}$ represent the linear effects of the independent variables $X_{1}$ and $X_{2}$, respectively, while $\beta_{11}$ and $\beta_{22}$ describe their quadratic effects and account for curvature in the response surface. The interaction coefficient $\beta_{12}$ represents the combined effect of simultaneous variations in $X_{1}$ and $X_{2}$ on the response. In the present study, $X_{1}$ and $X_{2}$ correspond to the critical method parameters, namely flow rate and mobile phase composition, respectively. Thus, the polynomial model enables quantitative evaluation of the individual, quadratic, and interactive effects of the investigated factors on the selected chromatographic responses.

The developed model was used to generate response surface and contour plots for visualizing factor effects. Numerical optimisation was performed using desirability function criteria to obtain optimal chromatographic conditions. Finally, the predicted optimal conditions were experimentally verified, confirming the validity and robustness of the method.

The chromatographic analysis was conducted under optimised analytical conditions using a high-performance liquid chromatography system equipped with a UV detector, quaternary pump, autosampler, and degassing unit. The separation was performed on a reversed-phase Orbit 100 C18 (150 × 4.6 mm; 3.5 µm) column, which provided effective resolution and peak symmetry for both analytes. According to the optimisation study using the Central Composite Design, the best phase composition of ACN: water (40:60 *v*/*v*) at a flow rate of 1.0 mL/min was selected as optimal. Injection volume was kept at 20 µL, and detection was at 240 nm because this wavelength provided sufficient sensitivity for both drugs. The mobile phase was filtered, freshly prepared, and degassed before use to provide a consistent baseline and reproducible results across the analysis. An all-inclusive control plan was followed in accordance with ICH guidelines to ensure method robustness and lifecycle management. The parameters of the critical method, such as flow rate and mobile phase composition, were kept within the MODR (Multidimensional Operable Design Region) limits. Intentional variations of ±0.1 mL/min in flow rate and ±2% in mobile phase composition did not result in any significant changes in retention time or peak characteristics, thereby validating the strength of the method. The system suitability parameters were always close to acceptable limits; the tailing factor was below 2.0; the theoretical plate count was greater than 2000; and the system suitability parameters were less than 2 per cent. These results indicate that the designed RP-HPLC method is highly robust and reproducible, and can be relied upon to provide routine analysis with a well-established control strategy.

### 2.5. Analytical Method Validation

The designed RP-HPLC procedure was validated using a lifecycle-based approach in line with ICH Q2(R2) guidelines. Linearity and range, accuracy, precision, sensitivity, robustness, and system suitability were the validation parameters. The validation strategy was developed based on the ATP and MODR values obtained during method development, ensuring consistent performance throughout its lifecycle. System suitability requirements were also correlated with pharmacopoeial recommendations [21].

#### 2.5.1. Linearity and Range

The optimised chromatographic conditions were used to establish the linearity of the method by analysing a series of standard solutions of Acyclovir (2–10 µg/mL) and Doxepin HCl (12–28 µg/mL). Least-squares linear regression was used to construct calibration curves by plotting peak area versus concentration. To test the goodness of fit of a model, the correlation coefficient (R^2^) and the analysis of residuals were used to verify the linearity and homoscedasticity. The concentration ranges selected were due to the desired analytical use, and it was found to be suitable for routine quantitative analysis, including lower and higher working concentrations in accordance with regulatory expectations [22].

#### 2.5.2. Precision

The method precision was assessed relative to the repeatability (intra-day precision) and intermediate precision (inter-day precision). Each analyte was selected at three concentrations within the linear range (low, medium, and high). The intra-day precision was determined by analysing each concentration in triplicate under the same operating conditions, and the inter-day precision was determined by repeating the procedure on different days. The findings were represented as the %RSD, which is the reproducibility of the method [23].

#### 2.5.3. Accuracy

The recovery studies at three levels (usually 80, 100, and 120% of the target concentration) were conducted to assess the method’s accuracy. Pre-analysed samples were pre-treated with known amounts of standard drug, and the mixtures were analysed under optimised chromatographic conditions. Each level was triply analysed, and percentage recovery as well as percentage RSD were calculated to determine the trueness of the method.

#### 2.5.4. Method Sensitivity

Sensitivity of the method was determined by calculating the limit of detection (*LOD*) and limit of quantitation (*LOQ*) using the standard deviation of response and slope of the calibration curve. The calculation of the *LOD* and *LOQ* was done based on the standard deviation of the response and the slope of the calibration curve using the following equations:

(1) $$LOD=3.3\times \frac{\sigma }{S}$$

(2) $$LOQ=10\times \frac{\sigma }{S}$$

where *σ* represents the standard deviation of the response, and *S* is the slope of the calibration curve. These parameters indicate the sensitivity of the developed method.

#### 2.5.5. Robustness

The developed RP-HPLC method was tested to assess its robustness, specifically its reliability under small, intentional changes in chromatographic parameters, as required by ICH Q2(R2). The critical method parameters (CMPs), such as flow rate (±0.1 mL/min), mobile phase composition (±2% organic phase), and detection wavelength (±2 nm), were varied around the optimised conditions. Standard solutions of Acyclovir and Doxepin hydrochloride at a given concentration in each variation were analysed in triplicate, and the effect of these changes on chromatographic responses, such as peak area, retention time, and tailing factor, was observed. The results were presented as mean, standard deviation (SD), and percentage relative standard deviation (%RSD), and compared to predefined acceptance criteria to determine method robustness.

#### 2.5.6. System Specificity

The system specificity of the developed analytical method was tested to ensure that the presence of solvents, excipients, or other interfering components did not influence the analyte response. The reported method involved blanking solutions of various solvents, including water and acetonitrile, and injecting them into the analytical system to ensure no interference at the drug’s retention time or at λmax. Moreover, a placebo formulation (cream base without a drug) and blank mobile phase were also analysed under the same experimental conditions. No interfering peaks or absorbance were observed at the selected analytical wavelength/retention time of the drug, indicating that excipients and solvents did not interfere with its detection. Accordingly, the procedure was discovered to be precise and applicable to the precise estimation of the drug in the cream formulation [24].

### 2.6. Preparation of Pharmaceutical Cream

A topical cream formulation containing Acyclovir and Doxepin hydrochloride (2.5% *w/v* each) was prepared using a conventional fusion method to ensure uniform drug distribution and physicochemical stability. Initially, the oil phase consisting of white petroleum jelly, white beeswax, and stearic acid was accurately weighed and melted together at a controlled temperature (70–75 °C) to obtain a homogeneous lipid base. In parallel, the aqueous phase was prepared by dissolving methyl paraben in purified water, followed by the addition of propylene glycol as a humectant and solubilizing agent. Tween 80 and Span 80 were incorporated as emulsifying agents to stabilize the oil-in-water system, while titanium dioxide was added as an opacifying agent. The aqueous phase was heated to the same temperature as the oil phase, then slowly incorporated into the oil phase under continuous stirring to form a uniform emulsion. After emulsification, the mixture was allowed to cool gradually while stirring continuously to prevent phase separation and ensure proper consistency. The accurately weighed quantities of acyclovir and doxepin HCl were incorporated into the base during the cooling phase to prevent thermal degradation, and the mixture was thoroughly mixed to achieve a uniform and stable cream formulation [25].

### 2.7. Quantification of the Drug

To perform quantitative analysis, an effective extraction process was used to recover both drugs from the semisolid matrix with minimal interference from excipients. A 100 mL volumetric flask was weighed, then 100 mg of total drug content was transferred (precisely weighed) into the volumetric flask. Methanol was chosen as the extraction solvent because it has a high solubilising capacity for both acyclovir and doxepin HCl, and they are compatible with RP-HPLC analysis. The methanol (10 mL) was then added to aid dispersion of the cream matrix and mixed thoroughly to break the emulsion. Methanol was then added up to the mark to ensure that the active pharmaceutical ingredients were fully solubilised. The mixture was then sonicated for 30 min to enhance the extraction efficiency by disrupting the lipidic base and facilitating the complete release of the drug into the solvent phase. Upon sonication, the solution was filtered through a 0.45 µm membrane filter to remove undissolved excipients and particulate matter, yielding a clear filtrate suitable for chromatographic analysis. This process provided a maximum recovery of both drugs with minimum interference of the matrix, thus enhancing the accuracy and reliability of the analytical method [26,27].

### 2.8. In-Vitro Drug Release

In-vitro drug release of the prepared cream formulation was determined using the dialysis bag diffusion method. An accurately weighed quantity of the cream formulation containing 2.5% *w/v* each of acyclovir and doxepin hydrochloride was placed in a 14 kDa MWCO dialysis membrane and tightly sealed. The dialysis bag was immersed in 100 mL phosphate buffer (pH 6.8) maintained at 37 ± 0.5 °C with continuous stirring at 100–150 rpm. Samples of 5 mL were withdrawn at predetermined intervals of 0.5, 1, 2, 3, 4, 6, 8, 12, and 24 h, and an equal volume of fresh phosphate buffer was added after each withdrawal to maintain sink conditions. The withdrawn samples (*n* = 3) were analyzed using the developed RP-HPLC method at 240 nm. The cumulative percentage drug release was calculated with correction for the volume of medium withdrawn during successive sampling [28].

## 3. Result

### 3.1. Selection of Analytical Wavelength

**Figure 3:**
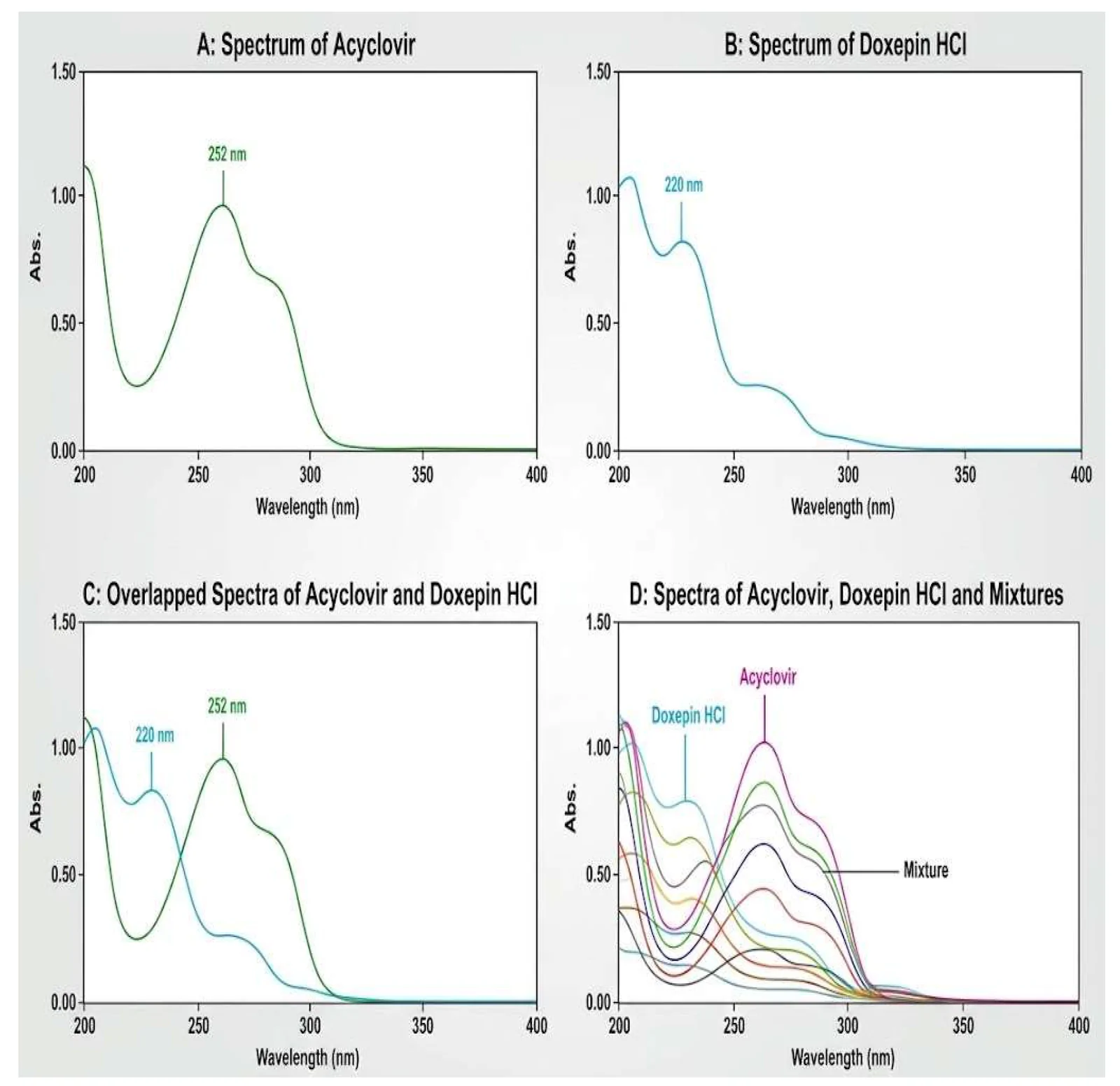
UV-Vis spectra profiles of Acyclovir, Doxepin HCl, and their combined solutions. (**A**) UV spectrum of Acyclovir (8 μg/mL) with $\lambda_{\max}$ at 252 nm. (**B**) UV spectrum of Doxepin HCl (4 μg/mL) with $\lambda_{\max}$ at 220 nm. (**C**) Overlapped UV absorption spectra displaying peak resolution for simultaneous estimation. (**D**) Zero-order overlay spectra of Acyclovir, Doxepin HCl, and synthetic laboratory mixtures across varying concentration ranges.

The selection of analytical wavelengths was based on UV–visible spectrophotometric analysis, and the corresponding spectral profiles are presented in Figure 3A–D. As shown in Figure 3A, Acyclovir exhibited a distinct and well-defined absorption maximum (λmax) at 252 nm, indicating adequate spectral sensitivity for quantitative analysis. Similarly, Figure 3B demonstrates the characteristic absorption maximum of Doxepin hydrochloride at 228 nm, confirming its distinct spectral behaviour. Figure 3C presents the overlapped absorption spectra of Acyclovir and Doxepin hydrochloride, highlighting their respective absorption maxima at 252 and 228 nm and demonstrating distinguishable spectral profiles of both analytes. Furthermore, Figure 3D illustrates the simultaneous spectral profiles of Acyclovir and Doxepin hydrochloride at different concentrations along with their combined mixture spectrum, confirming appreciable absorbance of both analytes within the selected wavelength region. Notably, both analytes exhibited simultaneous and adequate absorbance at 240 nm in the combined spectral profile without significant spectral interference, supporting the suitability of this wavelength for simultaneous detection. Thus, the spectral analysis provided a scientific basis for the selection of 240 nm as the detection wavelength for the subsequent RP-HPLC method development and simultaneous estimation of Acyclovir and Doxepin hydrochloride.

### 3.2. Optimisation Study

The statistical analysis of experimental data revealed that all responses, including retention time and tailing factor of both analytes, predominantly followed quadratic and interaction (2FI) models, as suggested by the fit summary. The detailed ANOVA and model adequacy statistics for the developed chromatographic models are demonstrated in Table 5. The developed models were highly significant, with *p*-values below 0.0001 for most responses, indicating a strong influence of the selected factors on chromatographic performance. The coefficient of determination (R^2^) values were 0.9988 for retention time (Acl), 0.9264 for tailing factor (Acl), 0.9947 for retention time (Dox), and 0.9144 for tailing factor (Dox), indicating excellent model fit and robustness. Furthermore, the predicted R^2^ values were in close agreement with the adjusted R^2^ values for most responses, confirming the models’ high predictive capability. The lack-of-fit was found to be non-significant for all responses (*p* > 0.05), indicating that the models adequately describe the experimental data. Adequate precision values were significantly greater than 4, confirming a sufficient signal-to-noise ratio and validating the models’ suitability for navigating the design space and optimising chromatographic conditions.

Second-order polynomial equations in terms of coded factors were generated to describe the relationship between the independent variables and the responses.

Retention Time (Acl) (Y_1_):

Y_1_ = 2.59 − 0.2027A − 0.3335B − 0.0757AB + 0.0753A^2^ + 0.0068B^2^

Tailing Factor (Acl) (Y_2_):

Y_2_ = 1.32 − 0.0500A − 0.0150B + 0.0000AB − 0.0862A^2^ + 0.0188B^2^

Retention Time (Dox) (Y_3_):

Y_3_ = 4.66 − 0.0305A − 0.1208B + 0.2200AB

Tailing Factor (Dox) (Y_4_):

Y_4_ = 1.18 + 0.0183A − 0.0050B + 0.0200AB − 0.0429A^2^ + 0.0271B^2^

The analysis of polynomial equations revealed that individual factors significantly influenced all chromatographic responses. Flow rate (Factor A (β1)) showed negative correlations with the retention times of both Acl (β1 = −0.2027) and Dox (β1 = −0.0305), indicating that higher flow rates elute both Acl and Dox faster. It also showed a negative influence on the tailing factor of Acl (1 = −0.0500), indicating an improvement in peak asymmetry at higher levels, whereas the tailing factor of Dox (1 = +0.0183) was slightly positively influenced. Conversely, mobile phase ratio (Factor B(β2)) showed a strong negative effect on the retention times of Acl (2 = −0.3335) and Dox (2 = −0.1208), which is consistent with reduced retention at increased organic composition. It also showed a small negative influence on tailing factors, indicating a slight improvement in peak symmetry. The combined and nonlinear behaviour of the system was additionally explained by the interaction (AB) and quadratic terms. The interaction effect was negative with the retention time of Acl (12 = −0.0757) and positive with the retention time of Dox (12 = +0.2200), implying a combined negative interaction effect between factors. In the case of tailing factors, the interaction term had a positive contribution, indicating a combined effect on peak symmetry. The quadratic terms verified the curvature in the response surface, especially in flow rate in retention time of Acl (β11 = +0.0753) and tailing factor of Acl (β11 = −0.0862), which implies nonlinear behaviour at extreme levels. On the same note, quadratic effects were also found on the Dox tailing factor, which proved to be a curved system response.

In conclusion, the flow rate had an overwhelmingly negative correlation with the retention time and tailing factor of Acl, and a mixed effect on Dox responses. Mobile phase ratio, on the other hand, showed a steadily negative correlation with retention time, implying decreased retention with increasing organic phase composition. These observations show that the flow rate is the main factor that determines the elution speed and peak symmetry, and that mobile phase composition is also a key factor used to control retention behaviour. Hence, a desirable compromise between the two aspects is necessary to obtain desirable chromatographic performance and effective separation.

### 3.3. Contour Analysis

**Figure 4:**
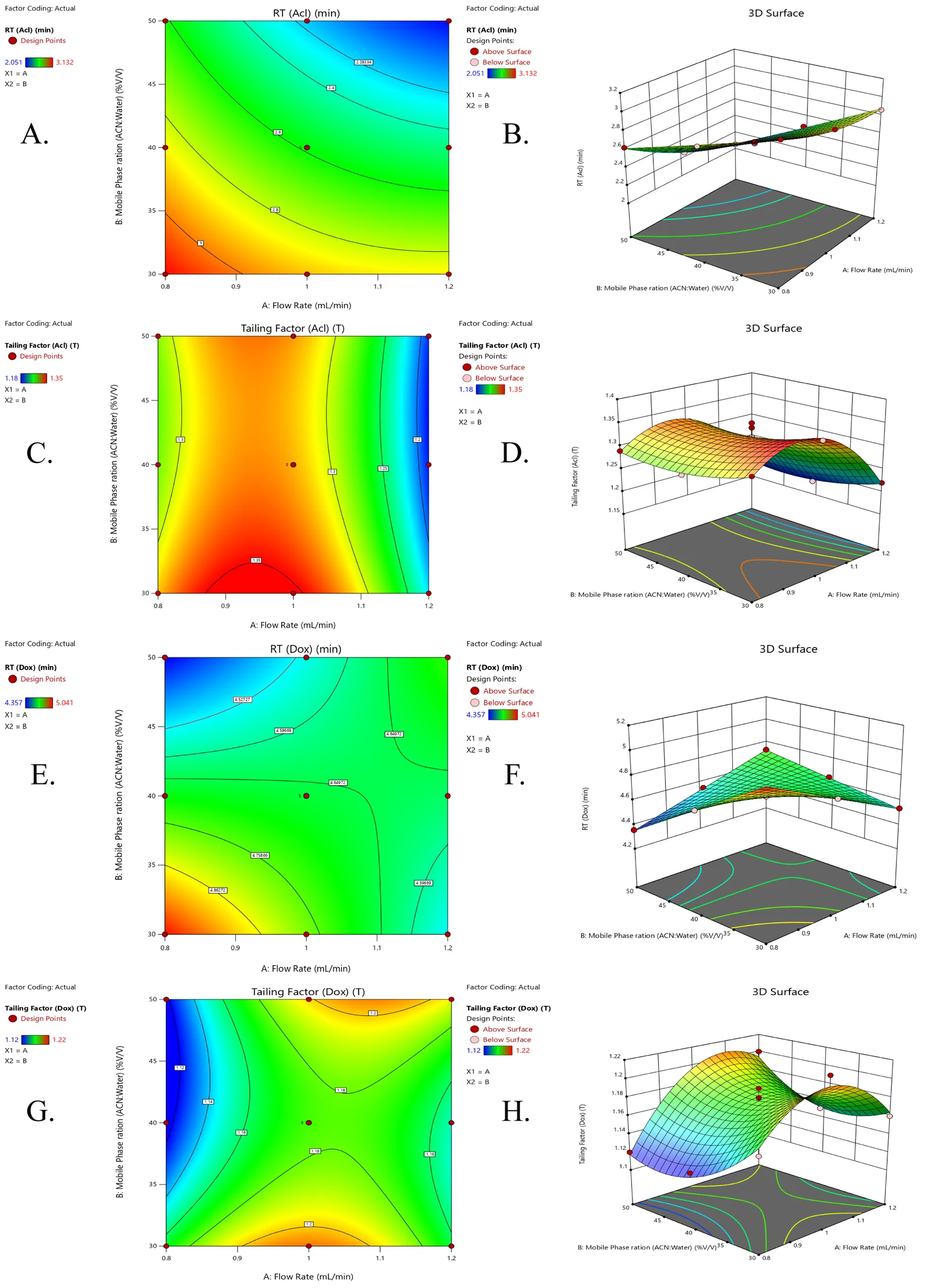
2D contour plots and corresponding three-dimensional (3D) response surface plots illustrating the combined effects of flow rate (Factor A) and mobile phase ratio (Factor B) on the chromatographic responses: (**A**,**B**) retention time of Acyclovir, (**C**,**D**) tailing factor of Acyclovir, (**E**,**F**) retention time of Doxepin HCl, and (**G**,**H**) tailing factor of Doxepin HCl.

To assess the effect of the interaction between flow rate (Factor A) and mobile phase ratio (Factor B) on the selected chromatographic responses based on QbD data, the contour plots and corresponding response surface plots were generated. The contour plot of retention time (Acl), shown in Figure 4A, exhibited an elliptical shape, indicating a significant interaction between the variables. The regression coefficients (β1 = −0.2027; β2 = −0.3335) indicate that retention time decreased with increasing flow rate and, more significantly, with increasing mobile phase ratio. The curvature in the contour plot was another indication that the levels of the two factors (A2 and B2) have significant quadratic effects on analyte retention. The corresponding 3D response surface (Figure 4B) corroborated this relationship by clearly demonstrating the combined influence of both factors on the retention behaviour of Acyclovir across the experimental domain.

The tailing factor (Acl) contour plot (Figure 4C) displayed a moderately curved surface with widely spaced contour lines, indicating a moderate influence of both factors. The tailing factor decreased with increasing flow rate (1 = −0.05 00), which was anticipated to result in enhanced peak symmetry, whereas the mobile phase ratio factor was relatively less pronounced (2 = −0.01 50). This plot curvature is consistent with the large quadratic term (A2), confirming that peak symmetry as a function of flow rate is nonlinear. The almost elliptical contour pattern indicates that flow rate is a dominant factor, with a limited interaction effect. The corresponding 3D response surface (Figure 4D) further demonstrated the curvature of the response and confirmed that an appropriate combination of flow rate and mobile phase composition was required to achieve acceptable peak symmetry for Acyclovir.

At the case of retention time (Dox) (Figure 4E), the contour plot presented a sharp elliptical shape, indicating that there was a strong interaction between flow rate and mobile-phase ratio. The retention time decreased with increasing flow rate (β1 = −0.0305) and mobile phase ratio (β2 = −0.1208), and the interaction term (β12 = +0.2200) indicated a significant synergistic effect between the two variables. The retention time was also highly sensitive to minor changes in both factors, as indicated by the close spacing of the contour lines. The 3D response surface depicted in Figure 4F further corroborates this interaction by illustrating a curved response profile and emphasizing the joint effect of flow rate and mobile phase composition on the retention of Doxepin HCl.

The contour plot of the tailing factor (Dox) (Figure 4G) showed a curved surface with moderate gradients, indicating the joint influence of both variables. The tailing factor slightly increased with flow rate (β1 = +0.0183) and decreased with mobile phase ratio (β2 = −0.0050). Large quadratic terms (A2 and B2) confirmed that there is curvature in the response surface, and the interaction term (β12 = +0.0200) confirmed that the simultaneous variation in both factors affects the shape of the peak. This three-dimensional response surface (Figure 4H) further demonstrated the nonlinear behavior and showed that the tailing factor was affected by the combined variation in both chromatographic parameters. An intermediate region within the experimental domain provided comparatively favourable tailing factor values, supporting the selection of optimized chromatographic conditions for achieving acceptable peak symmetry.

### 3.4. Design Space

The design space was established using the developed models to identify the optimal region in which all responses met the predefined criteria. The numerical optimisation indicated that a flow rate of approximately 0.95–1.1 mL/min and a mobile phase ratio of 35–45% ACN provided the most desirable chromatographic performance. Within this region, the predicted responses of Acl and Dox viz retention time and tailing factor are within the acceptable limit. The overlay plot confirmed that this region represents a robust design space in which small variations in factors do not significantly affect the responses. It was observed that increasing the flow rate beyond 1.2 mL/min led to a sharp decrease in retention time, while lower flow rates resulted in longer run times. Similarly, higher mobile phase ratios led to faster elution but could affect peak symmetry and separation. Therefore, the selected design space balances retention behaviour, peak symmetry, and method robustness, confirming the reliability of the optimised chromatographic method.

**Figure 5:**
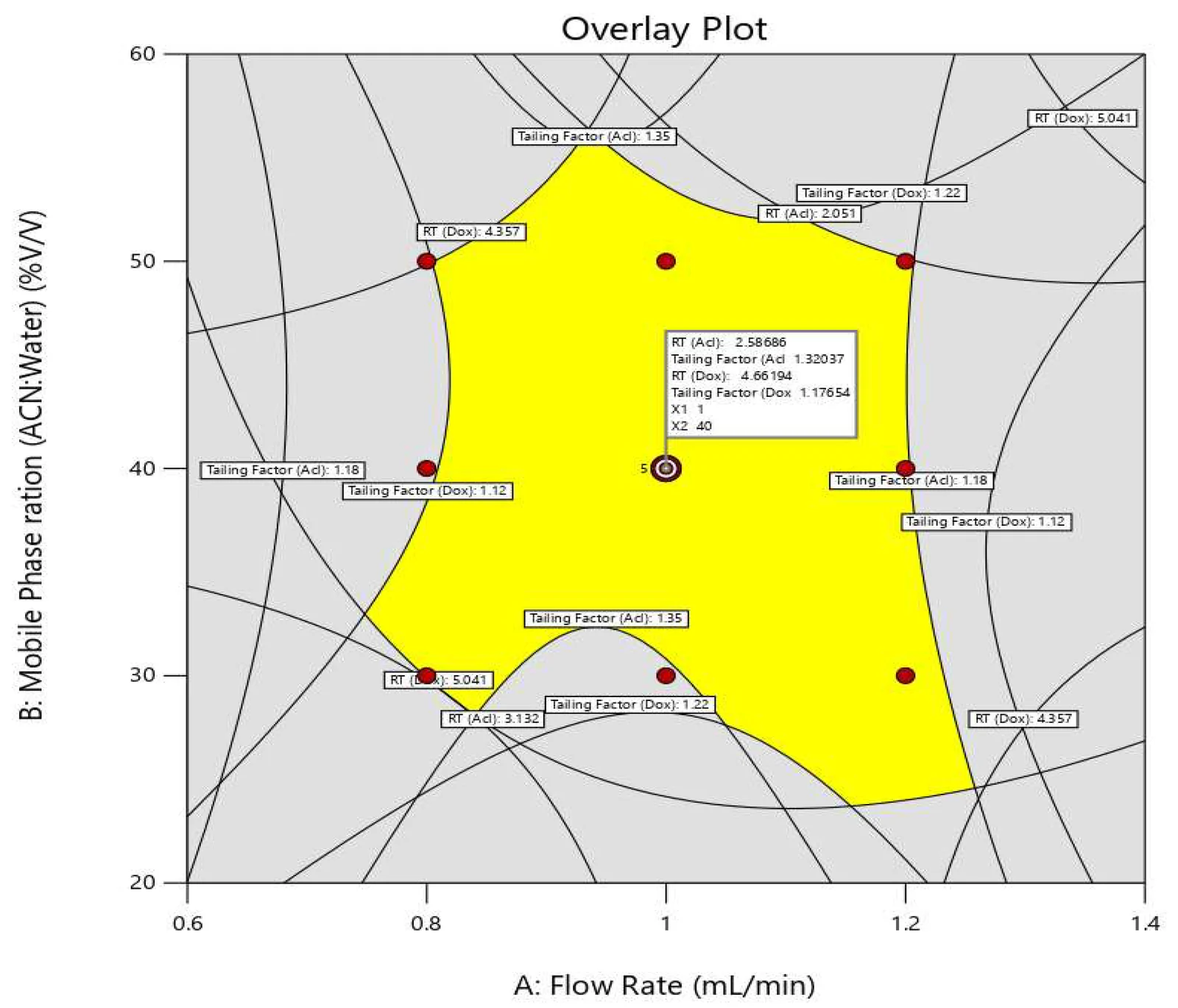
Design space for the developed HPLC method.

As depicted in Figure 5, the overlay plot delineates the MODR, wherein the yellow-shaded area represents the multidimensional design space satisfying the predefined acceptance criteria for all critical analytical responses, while the surrounding grey region denotes conditions associated with failure to meet one or more response constraints. The optimized operating point (1.0 mL/min flow rate and 40% ACN) yielded predicted retention times of 2.5866 and 4.6619 min for Acyclovir and Doxepin HCl, respectively, with corresponding tailing factors of 1.3037 and 1.1765. The positioning of the optimized condition within the established design space indicates an adequate robustness margin and confirms that minor variations in the critical method parameters are unlikely to adversely influence chromatographic performance.

### 3.5. Numerical Validation of the Optimization Model

Numerical validation of the optimization model demonstrated close agreement between predicted and experimental responses. The percentage bias ranged from −1.734 to +1.834% for ACV retention time, −2.308 to +1.810% for ACV tailing factor, −1.321 to +1.353% for DOX retention time, and −2.377 to +2.652% for DOX tailing factor. For the selected F5 formulation, the corresponding biases were +1.058%, +1.617%, +0.301%, and −0.679%, respectively, confirming close numerical agreement between the predicted and experimental responses (Table 6).

### 3.6. Optimised Chromatographic Conditions

**Figure 6:**
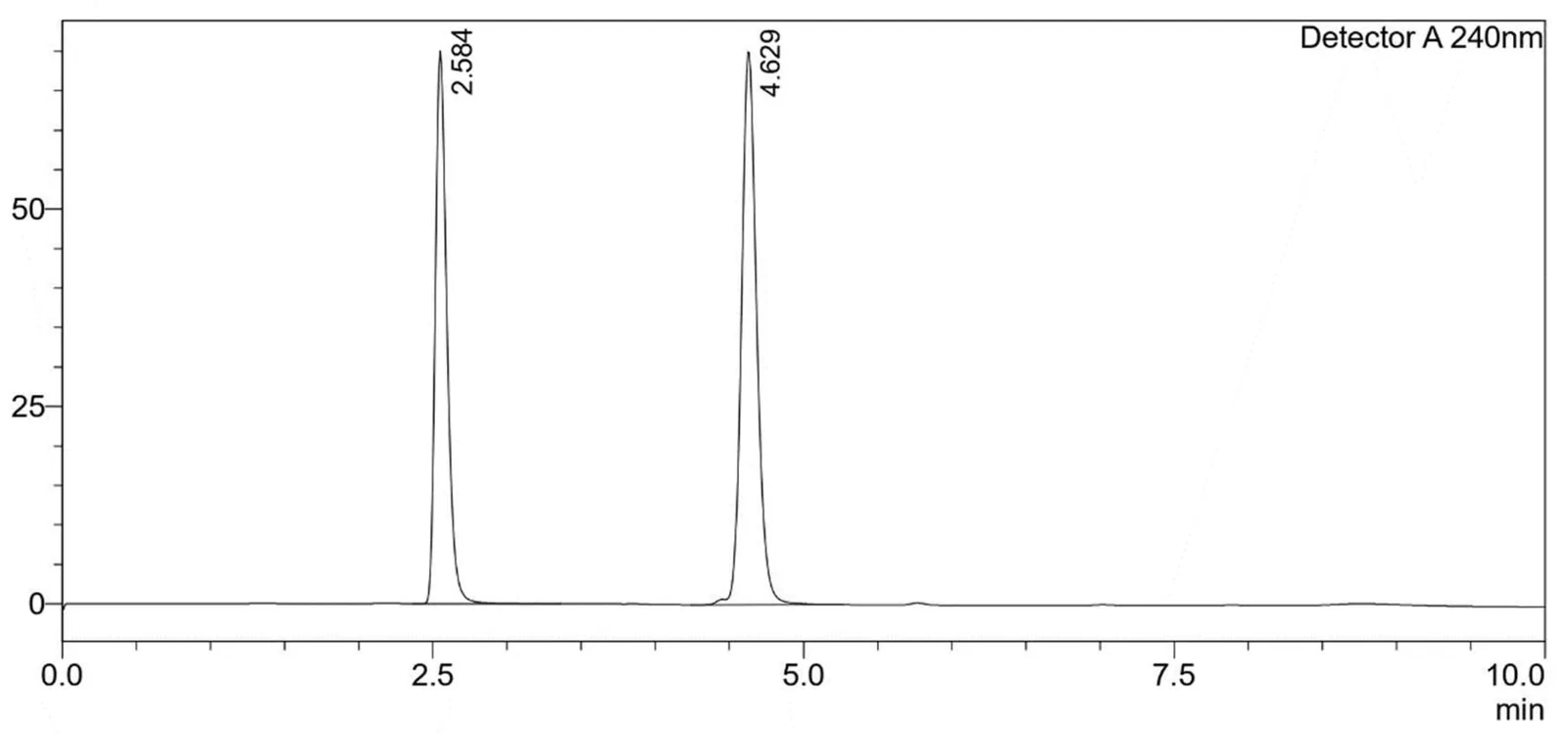
Optimised chromatogram of mixed standard solution containing 10 µg/mL Acyclovir and 16 µg/mL Doxepin HCl at wavelength 240 nm.

The chromatographic conditions were optimised through systematic trials by varying the mobile-phase composition and detection wavelength to achieve efficient separation with acceptable peak symmetry. Initial trials with lower proportions of the organic phase resulted in increased retention times, peak broadening, and unsatisfactory tailing factors. However, optimisation led to the selection of an isocratic mobile phase consisting of ACN: water (40:60 *v*/*v*) at a flow rate of 1.0 mL/min, with detection at 240 nm. Under these conditions, both analytes exhibited well-resolved, sharp, and symmetrical peaks within a short analysis time. The retention times were approximately 2.58 min for Acyclovir and 4.64 min for Doxepin hydrochloride, with tailing factors less than 2, indicating good peak symmetry, as shown in Figure 6. The method demonstrated a stable baseline, reproducible retention behaviour, and satisfactory system suitability parameters, confirming that the selected chromatographic conditions are optimal for the simultaneous estimation of both drugs.

### 3.7. Analytical Method Validation Results (RP-HPLC)

#### 3.7.1. System Suitability

The system suitability parameters obtained under optimised chromatographic conditions were within the predefined acceptance limits, confirming the proper functioning of the chromatographic system. Key parameters, including retention time, tailing factor, and theoretical plate count, showed consistent, reproducible performance, with tailing factors below 2 and theoretical plates exceeding 2000 for both analytes. These results indicate good peak symmetry and column efficiency. System suitability evaluation is essential to ensure that the chromatographic system maintains performance during analysis, as prolonged operation may affect system reliability and data quality. Furthermore, the method’s sensitivity, as indicated by the LOD and LOQ values, confirmed its ability to detect and quantify analytes at low concentrations. Overall, the system suitability results in Table 7 validate the robustness, reliability, and consistency of the developed RP-HPLC method, supporting its applicability for routine analytical use.

#### 3.7.2. Linearity and Range

**Figure 7:**
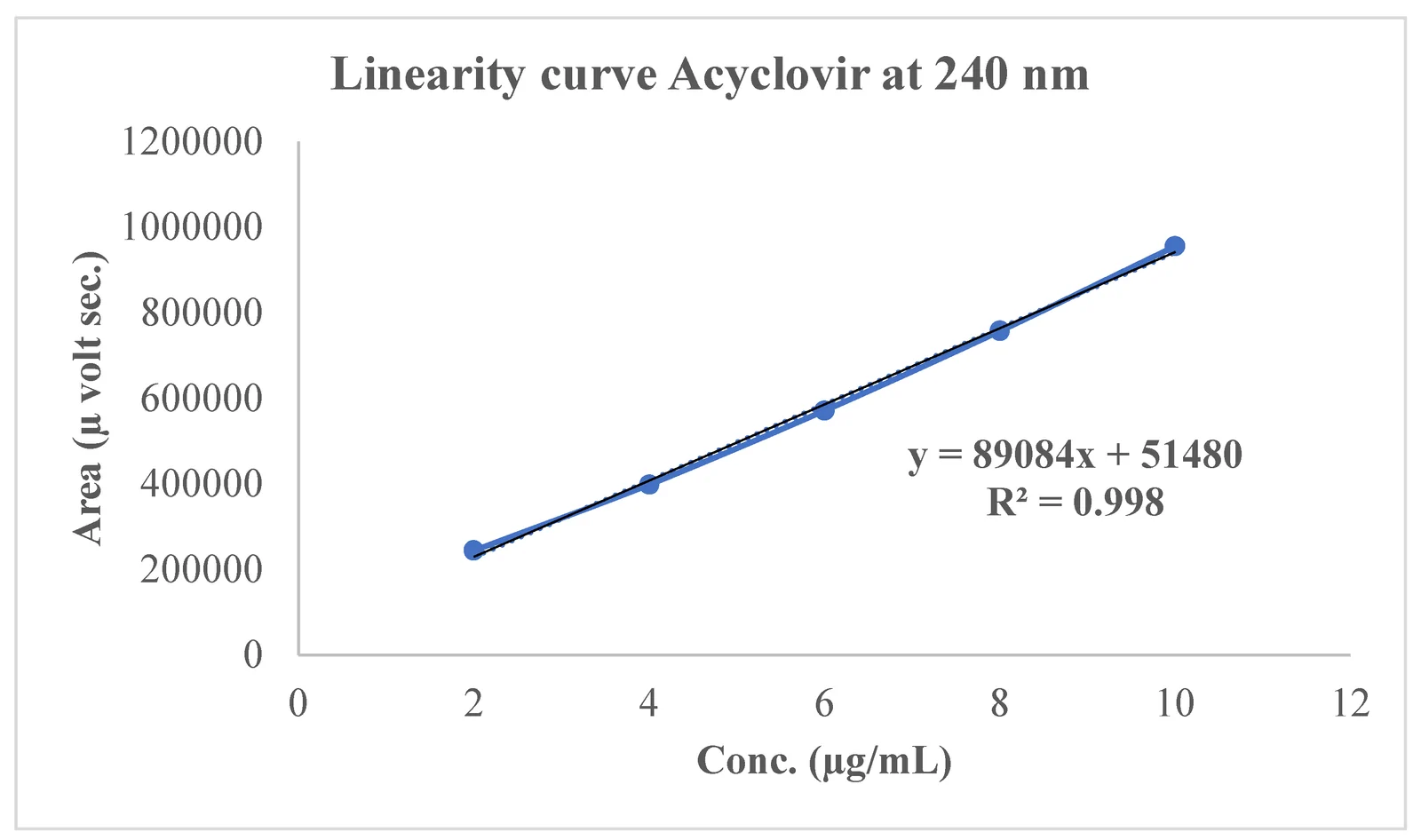
Linearity curve of Acyclovir.

**Figure 8:**
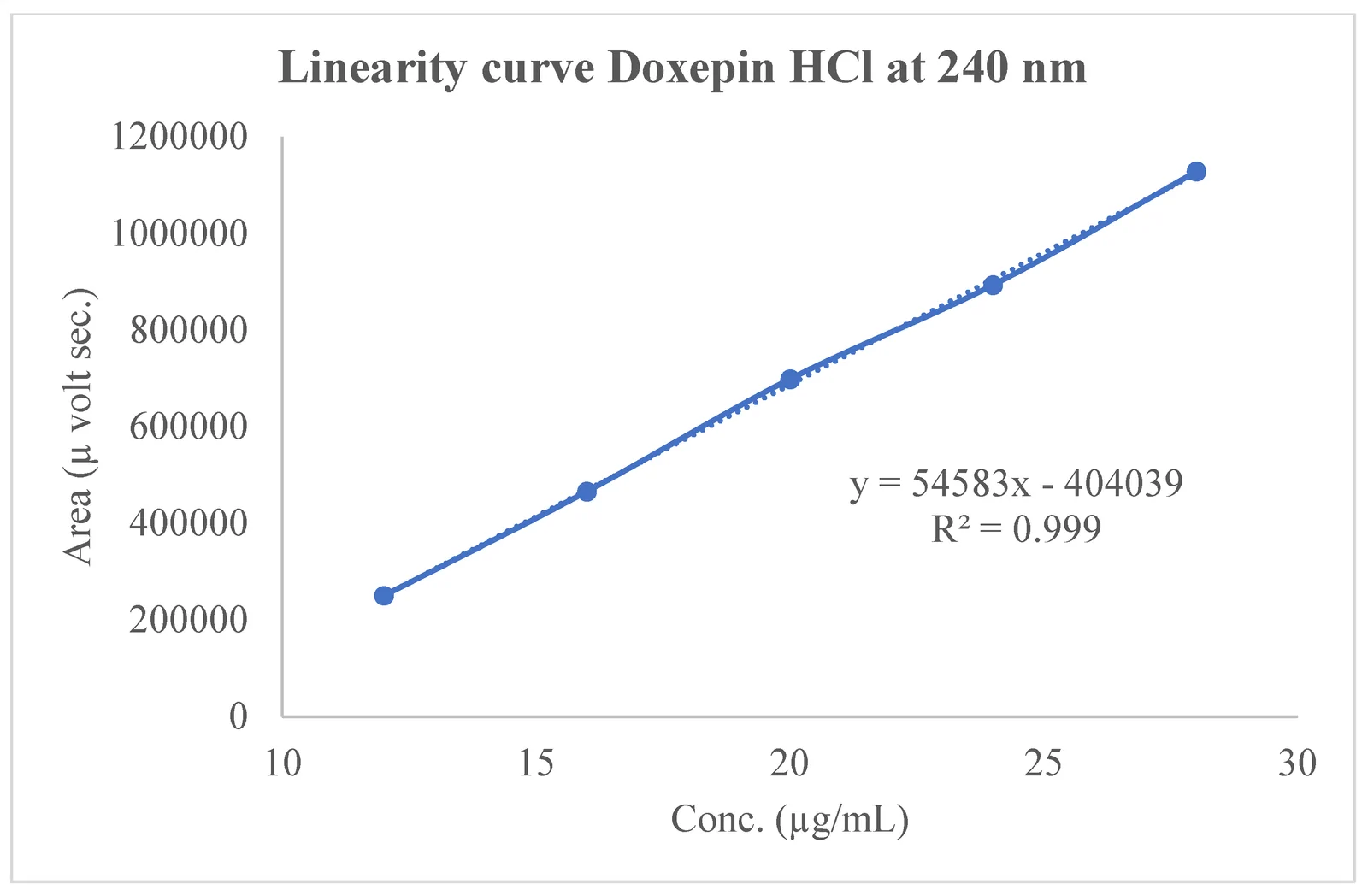
Linearity of Doxepin HCl.

The developed RP-HPLC method demonstrated excellent linearity over the concentration ranges of 2–10 µg/mL for Acyclovir and 12–28 µg/mL for Doxepin hydrochloride. The calibration curves, plotted as peak area against concentration, showed a strong linear relationship, with correlation coefficients of R^2^ = 0.998 for Acyclovir and 0.999 for Doxepin HCl, as shown in Figure 7 and Figure 8. However, the predefined ATP criterion of R^2^ ≥ 0.999 was achieved for doxepin HCl but was not achieved for acyclovir. But the peak area increased proportionally with concentration, confirming the suitability of the selected ranges for quantitative analysis. The residuals were minimal, indicating good model fit and homoscedasticity across the studied concentration ranges.

#### 3.7.3. Precision

The precision of the method was evaluated in terms of intra-day and inter-day variability at low, medium, and high concentration levels. The Relative Standard Deviation (RSD) values for intra-day precision were 0.08–0.41% for Acyclovir and 0.12–0.17% for Doxepin HCl, indicating excellent repeatability. Similarly, inter-day precision studies conducted over three consecutive days yielded %RSD values below 0.5% for both analytes, confirming the method’s reproducibility under normal operating conditions. Table 8 summarizes the precision study results for the developed HPLC method for both drugs.

#### 3.7.4. Accuracy

The accuracy of the method was determined using recovery studies at different concentration levels. The percentage recovery for Acyclovir ranged from 99.39% to 101.38%, while for Doxepin HCl it ranged from 99.56% to 100.35%, as listed in Table 9. The low variability in recovery values, along with minimal %RSD, confirmed the trueness and reliability of the developed method, indicating the absence of interference from other components.

#### 3.7.5. Method Sensitivity (LOD and LOQ)

The method’s sensitivity was evaluated by determining the LOD and LOQ. The LOD values were 0.0217 µg/mL for Acyclovir and 0.0656 µg/mL for Doxepin HCl, while the LOQ values were 0.0259 µg/mL and 0.0784 µg/mL, respectively. These results indicate that the developed RP-HPLC method is sufficiently sensitive for the detection and quantification of both analytes at low concentrations.

#### 3.7.6. Robustness

The robustness of the developed RP-HPLC method was confirmed by evaluating the effect of small, deliberate variations in chromatographic conditions, as summarized in Table 10. The results demonstrated that minor changes in flow rate, mobile phase composition, and detection wavelength had no significant impact on chromatographic performance. The observed mean concentration, SD, and %RSD remained within acceptable limits, with %RSD consistently below 2% for both analytes. These findings indicate that the developed method is highly robust and capable of producing reliable and reproducible results under slightly varied analytical conditions.

#### 3.7.7. Specificity Study

**Figure 9:**
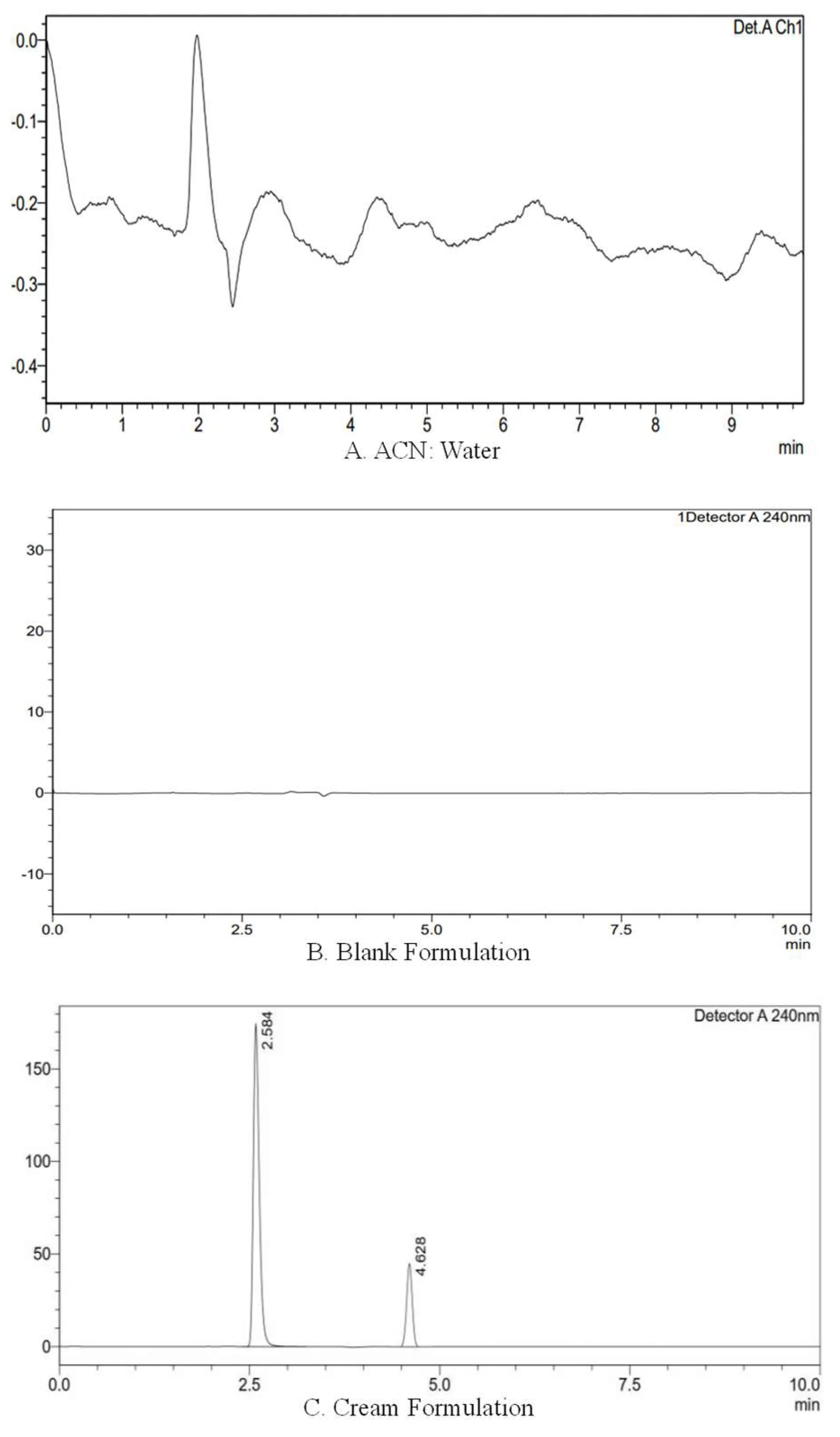
System specificity data. (**A**) ACN: Water HPLC solvent, (**B**) Blank formulation with all the excipients without drugs, (**C**) Pharmaceutical cream formulation containing Acyclovir and Doxepin HCl.

The specificity test indicated that the developed analytical method was potentially useful for measuring the drug in the presence of formulation excipients. To determine whether any interference occurred at the 240 nm wavelength analysis, blank solutions of different solvents, including methanol, water and ACN, were analysed under the same experimental conditions. There were no major absorbance or interfering peaks at 240 nm with the drug, and so no solvent interference was observed. Further, a placebo formulation (cream base, no drug) and a blank medium were also analysed, and neither showed any interference at 240 nm. The method’s selectivity and its ability to determine drug content in the cream formulation, as shown in Figure 9, were validated by the results obtained.

### 3.8. Drug Content

Both Acyclovir and Doxepin HCl were evaluated for drug content in the prepared cream formulation, and the results were satisfactory in terms of conformity and drug distribution accuracy. For Acyclovir, the drug content of the product was recorded to be within the range of 99.3% to 100.5%, with a mean drug content of 99.71% and a percentage RSD of +/−0.501, indicating no significant variation between replicates as listed in Table 11. Similarly, for Doxepin HCl, the drug content percentage was determined to be between 99.1% and 100.8%, with a mean of 99.86% and a standard deviation of 1.604. The low percentage standard deviation values (0.02) for the two drugs suggest that the method has good precision and reproducibility. These results confirm that the prepared cream formulation exhibits good homogeneity in drug distribution and is in line with the desired pharmaceutical standards for drug content.

### 3.9. In-Vitro Release

**Figure 10:**
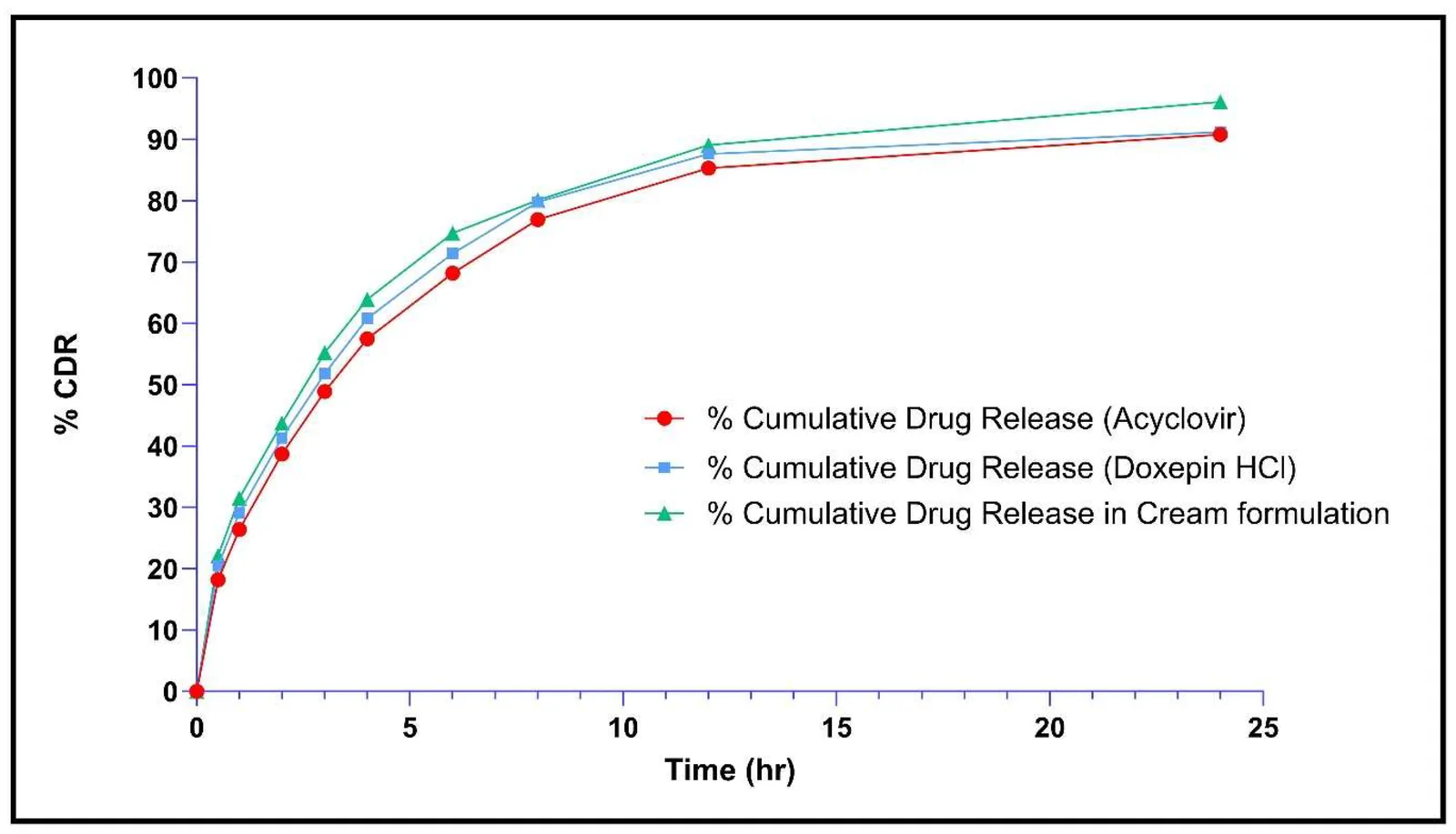
In-vitro drug release of the developed cream formulation.

The in vitro drug-release profile of Acyclovir, Doxepin HCl, and the optimised cream formulation was evaluated using the dialysis-bag diffusion method, and the cumulative percentage drug release was determined over 24 h. As shown in Figure 10, both pure drugs exhibited a slower release than the cream formulation. The cream formulation showed an initial burst release of 22.1% within 0.5 h, followed by sustained, controlled release, reaching 96.1% at 24 h. In comparison, Acyclovir and Doxepin HCl showed 90.8% and 91.2% drug release, respectively, after 24 h.

## 4. Conclusions

The present study successfully developed and validated a robust, sensitive, and reliable RP-HPLC method for the simultaneous estimation of Acyclovir and Doxepin hydrochloride in bulk and cream formulations using an Analytical Quality by Design (AQbD) approach. The systematic application of risk assessment tools, including the Ishikawa diagram and risk matrix, enabled the identification of critical method parameters, while Central Composite Design (CCD) facilitated efficient optimisation of chromatographic conditions. The optimised method demonstrated excellent system suitability, with well-resolved peaks, acceptable retention times, and good peak symmetry within a short analysis time. Validation studies confirmed that the method is linear, accurate, precise, and sensitive, with %RSD values within acceptable limits and high correlation coefficients, indicating strong reliability for quantitative analysis. Furthermore, the method exhibited good robustness against small deliberate variations in analytical conditions, ensuring consistent performance. The developed HPLC method was used to quantitatively determine the concentrations of acyclovir and doxepin hydrochloride in the developed cream formulation. Overall, the proposed AQbD-based RP-HPLC method provides a simple, rapid, and cost-effective analytical tool suitable for routine quality control of combination drug products containing Acyclovir and Doxepin HCl.

## Figures and Tables

**Table 1:** Analytical Target Profile for RP-HPLC Method.

ATP Elements	Target Criteria	Justification
Analytical Technique	RP-HPLC	Suitable for simultaneous estimation with high sensitivity and specificity
Analytes	Acyclovir and Doxepin hydrochloride	Combination therapy requires simultaneous estimation
Dosage Form	Cream formulation	A complex matrix requires a selective method
Purpose	Quantitative assay	Required for quality control and regulatory compliance
Specificity	No interference from excipients	Ensures HPLC method specificity
Accuracy	98–102% recovery	As per ICH Q2(R2) acceptance criteria
Precision	%RSD < 2%	Ensures reproducibility
Linearity	r^2^ ≥ 0.999	Required for reliable quantification
Range	80–120% of test concentration	As per ICH Q2(R2) recommendations
Resolution	>2 between peaks	Ensures proper separation
Tailing Factor	<2	Indicates good peak symmetry
Theoretical Plates	>2000	Indicates column efficiency
Analysis Time	<10 min	Ensures rapid analysis

**Table 2:** Risk evaluation of critical method variables affecting ATP.

ATP Requirement	CQA	CMA	CPP	Severity (Impact)	Optimization Required	Justification
Accurate quantification	Peak area response	Solvent purity, mobile phase composition	Injection volume, wavelength	High	Yes	Directly affects assay accuracy and reliability
High specificity	Resolution between peaks	Mobile phase ratio, column chemistry	Flow rate, temperature	High	Yes	Critical for the separation of analytes and impurities
Precision (%RSD < 2%)	Peak area reproducibility	Sample prep consistency	Injection volume, flow rate	Medium	Yes	Influences repeatability but is manageable with control
Run time	Retention time	Mobile phase composition	Flow rate	Medium	Yes	Impacts efficiency, but not critical to accuracy
Peak symmetry	Tailing factor	Column packing, pH	Flow rate	Medium	Yes	Affects integration and quantification
Column efficiency	Theoretical plates	Column type	Flow rate, temperature	Low	No	Important, but less critical if the minimum criteria are met
Linearity	Peak area vs. conc.	Standard preparation	Injection volume	Low	No	Usually controlled by calibration

**Table 3:** Risk matrix for developed HPLC method.

Parameter	Retention Time	Peak Area	Tailing Factor	Theoretical Plates
Mobile phase composition				
Flow rate				
Detection wavelength				
Column type (C18, particle size)				
Injection volume				
Column temperature				
Sample preparation				

**Table 4:** CCD model experiment for optimisation study.

Run	Flow Rate	Mobile Phase Ratio (ACN: Water)	RT (Acl)	Tailing Factor (Acl)	RT (Dox)	Tailing Factor (Dox)
1	1	30	2.937	1.35	4.782	1.22
2	1	50	2.257	1.32	4.545	1.19
3	1.2	40	2.478	1.18	4.637	1.15
4	1	40	2.581	1.28	4.649	1.19
5	1	40	2.578	1.34	4.652	1.18
6	0.8	50	2.623	1.29	4.357	1.12
7	1.2	30	2.863	1.22	4.537	1.16
8	0.8	30	3.132	1.32	5.041	1.16
9	0.8	40	2.853	1.28	4.692	1.12
10	1.2	50	2.051	1.19	4.733	1.2
11	1	40	2.581	1.35	4.667	1.16
12	1	40	2.598	1.32	4.679	1.17
13	1	40	2.589	1.32	4.634	1.18

**Table 5:** Detailed ANOVA and model adequacy statistics for the developed chromatographic models.

Response	Model/Term			Sum of Squares		df		Mean Square		F-Value		*p*-Value
RT (ACV)	Model (Quadratic)			0.9565		5		0.1913		1165.49		<0.0001
	A—Flow rate			0.2464		1		0.2464		1501.46		<0.0001
	B—ACN: Water ratio			0.6673		1		0.6673		4065.76		<0.0001
	AB			0.0230		1		0.0230		139.84		<0.0001
	A^2^			0.0157		1		0.0157		95.52		<0.0001
	B^2^			0.0001		1		0.0001		0.7884		0.4041
	Residual			0.0011		7		0.0002		—		—
	Lack of fit			0.0009		3		0.0003		4.44		0.0918
	Pure error			0.0003		4		0.0001		—		—
	Cor. total			0.9576		12		—		—		—
Tailing factor (ACV)	Model (Quadratic)			0.0375		5		0.0075		17.63		0.0008
	A—Flow rate			0.0150		1		0.0150		35.24		0.0006
	B—ACN: Water ratio			0.0014		1		0.0014		3.17		0.1181
	AB			0.0000		1		0.0000		0.0000		1.0000
	A^2^			0.0205		1		0.0205		48.23		0.0002
	B^2^			0.0010		1		0.0010		2.29		0.1738
	Residual			0.0030		7		0.0004		—		—
	Lack of fit			0.0001		3		0.0000		0.0460		0.9851
	Pure error			0.0029		4		0.0007		—		—
	Cor. total			0.0405		12		—		—		—
RT (DOX)	Model (2FI)			0.2868		3		0.0956		563.34		<0.0001
	A—Flow rate			0.0056		1		0.0056		32.89		0.0003
	B—ACN: Water ratio			0.0876		1		0.0876		516.24		<0.0001
	AB			0.1936		1		0.1936		1140.87		<0.0001
	Residual			0.0015		9		0.0002		—		—
	Lack of fit			0.0003		5		0.0001		0.2192		0.9365
	Pure error			0.0012		4		0.0003		—		—
	Cor. total			0.2883		12		—		—		—
Tailing factor (DOX)	Model (Quadratic)			0.0092		5		0.0018		14.95		0.0013
	A—Flow rate			0.0020		1		0.0020		16.33		0.0049
	B—ACN: Water ratio			0.0002		1		0.0002		1.21		0.3068
	AB			0.0016		1		0.0016		12.96		0.0087
	A^2^			0.0051		1		0.0051		41.22		0.0004
	B^2^			0.0020		1		0.0020		16.39		0.0049
	Residual			0.0009		7		0.0001		—		—
	Lack of fit			0.0003		3		0.0001		0.8830		0.5216
	Pure error			0.0005		4		0.0001		—		—
	Cor. total			0.0101		12		—		—		—
Model adequacy statistics												
Response	Selected model	R^2^	Adjusted R^2^		Predicted R^2^		Adeq. Precision		Std. Dev.		CV (%)	
RT (ACV)	Quadratic	0.9988	0.9979		0.9904		123.2041		0.0128		0.4881	
Tailing factor (ACV)	Quadratic	0.9264	0.8739		0.8794		12.1293		0.0206		1.60	
RT (DOX)	2FI	0.9947	0.9929		0.9906		94.3363		0.0130		0.2794	
Tailing factor (DOX)	Quadratic	0.9144	0.8532		0.5810		12.3632		0.0111		0.9504	

**Table 6:** Validation of optimization results.

Response	Formulation	Predicted Value	Original Experimental Value	Original Bias (%)
ACV Retention Time (min)	F1	2.669	2.630	−1.461
	F2	2.563	2.610	+1.834
	F3	2.595	2.550	−1.734
	F4	2.560	2.600	+1.563
	F5	2.553	2.580	+1.058
ACV Tailing Factor	F1	1.328	1.350	+1.657
	F2	1.324	1.300	−1.813
	F3	1.326	1.350	+1.810
	F4	1.300	1.270	−2.308
	F5	1.299	1.320	+1.617
DOX Retention Time (min)	F1	4.692	4.630	−1.321
	F2	4.638	4.700	+1.337
	F3	4.646	4.610	−0.775
	F4	4.657	4.720	+1.353
	F5	4.656	4.670	+0.301
DOX Tailing Factor	F1	1.176	1.190	+1.190
	F2	1.172	1.160	−1.024
	F3	1.169	1.200	+2.652
	F4	1.178	1.150	−2.377
	F5	1.178	1.170	−0.679

**Table 7:** System Suitability Parameters of Developed RP-HPLC Method.

Parameter	Acyclovir	Doxepin
Retention Time (min)	2.58	4.67
Tailing Factor	1.32	1.17
Peak Area	956,290 ± 8329	466,296 ± 4185
Theoretical Plates	8300 ± 132	8600 ± 119
Resolution	2.09	
LOD (µg/mL)	0.0217	0.0259
LOQ (µg/mL)	0.0656	0.0784
Slope	89,084.45	1244.9
R^2^	0.998	0.999

**Table 8:** Experimental Precision study data.

Drug	Conc. (µg/mL)	Mean Peak Area	Intra-day %RSD	Inter-day %RSD (Day 1)	Inter-day %RSD (Day 2)	Inter-day %RSD (Day 3)
Acyclovir	6	571,740	0.41	0.46	0.10	0.32
	8	757,914	0.15	0.25	0.29	0.30
	10	956,306	0.08	0.13	0.14	0.29
Doxepin HCl	20	698,965	0.17	0.18	0.26	0.31
	24	893,704	0.12	0.24	0.16	0.28
	28	1,128,550	0.15	0.21	0.18	0.19

**Table 9:** Experimental accuracy data.

Drug	Level (%)	Amount Added (µg/mL)	Amount Found (µg/mL)	% Recovery	%RSD
Acyclovir	80%	4.0	4.02	100.50	0.41
	100%	5.0	5.03	100.60	0.32
	120%	6.0	5.96	99.39	0.28
Doxepin HCl	80%	16.0	15.93	99.56	0.17
	100%	20.0	20.07	100.35	0.14
	120%	24.0	23.92	99.67	0.12

**Table 10:** Robustness Study of RP-HPLC Method.

Parameter Variation	Drug	Mean Conc. Found (µg/mL)	SD (*n* = 3)	%RSD
Flow Rate (0.9 mL/min)	Acyclovir	5.94	±0.03	0.50
	Doxepin HCl	19.87	±0.04	0.20
Flow Rate (1.1 mL/min)	Acyclovir	6.02	±0.02	0.33
	Doxepin HCl	20.05	±0.03	0.15
Mobile Phase (38:62)	Acyclovir	5.96	±0.04	0.67
	Doxepin HCl	19.92	±0.05	0.25
Mobile Phase (42:58)	Acyclovir	6.04	±0.03	0.49
	Doxepin HCl	20.08	±0.04	0.20
Wavelength (238 nm)	Acyclovir	5.98	±0.02	0.33
	Doxepin HCl	19.95	±0.03	0.15
Wavelength (242 nm)	Acyclovir	6.01	±0.03	0.49
	Doxepin HCl	20.02	±0.02	0.10

**Table 11:** Assay of Acyclovir and Doxepin in Cream formulation.

Drug	Drug Present in Preparation Sample (µg/mL)	Amount Found (µg/Ml) Mean of Three Replicates (*n* = 3)	Mean Purity (%) Mean of Three Replicates (*n* = 3)	%RSD
Acyclovir	10	9.971	99.71	±0.501
Doxepin HCl	10	9.986	99.86	±0.604

## Data Availability

Data will be available on request to the corresponding authors.

## Abbreviations

AQbD	Analytical Quality by Design
ATP	Analytical Target Profile
CCD	Central Composite Design
CMPs	Critical Method Parameters
HPLC	High-Performance Liquid Chromatography
LOD	Limit of Detection
LOQ	Limit of Quantification
